# Provision of Salt at No Cost Does Not Affect Total Discretionary Salt Intakes among Adult Females in Ethiopia: Secondary Analysis of a Community-Based Intervention Trial

**DOI:** 10.1016/j.cdnut.2026.107710

**Published:** 2026-05-06

**Authors:** Debritu Nane, Mengistu Fereja, Isaac Agbemafle, Charles D Arnold, Meseret Woldeyohannes, Christine M McDonald, Masresha Tessema, Homero Martinez, Kenneth H Brown

**Affiliations:** 1 Department of Nutrition and Institute for Global Nutrition, University of California, Davis, CA, United States; 2 Department of Reproductive Health and Nutrition, School of Public Health, Wolaita Sodo University, Sodo, Ethiopia; 3 Nutrition, Environmental Health, and Non-Communicable Disease Research Directorate, Ethiopian Public Health Institute, Addis Ababa, Ethiopia; 4 Department of Nutrition, University of Rhode Island, Kingston, RI, United States; 5 Fred N. Binka School of Public Health, University of Health, and Allied Sciences, Ho, Ghana; 6 Division of Gastroenterology, Hepatology and Nutrition, Department of Pediatrics, University of California San Francisco, CA, United States; 7 Research and Development Unit, Nutrition International, Ottawa, ON, Canada

**Keywords:** discretionary salt intake, salt iodization, folic acid fortification, folate deficiency, Ethiopia

## Abstract

**Background:**

Ethiopia has near-universal access to iodized salt, and national authorities are considering adding folic acid to iodized salt to address folate insufficiency and neural tube defects. However, providing double-fortified salt containing both iodine and folic acid (DFS-IoFA) may increase discretionary salt intake.

**Objectives:**

To evaluate whether providing refined fortified salt at no cost affects discretionary salt intake among nonpregnant adult females in Oromia, Ethiopia.

**Methods:**

We conducted a secondary analysis of weighed food records (WFRs) collected during a blinded, randomized controlled trial of DFS-IoFA in which participants were blinded to the salt they received. Of the 360 trial participants, 342 completed WFRs during the intervention and were included in the analysis; 143 of these participants also completed WFRs before the intervention, enabling paired comparisons, whereas the remaining 199 were assessed only during the intervention period. Discretionary salt intakes before and during the intervention were compared using paired *t*-tests for participants with data at both time points, and independent *t*-tests were used to compare intakes across participant subgroups. Usual intake was estimated by using the National Cancer Institute method.

**Results:**

The mean observed discretionary salt intake among all participants during the intervention (*N* = 342) was 8.4 ± 3.8 g/d. Among the 143 females assessed both before and during the intervention, discretionary salt intake was 8.7 ± 4.3 g/d before and 8.8 ± 4.0 g/d during the intervention (*P* = 0.8). Preintervention intake in this group did not differ from the mean intake of all participants during the intervention (*P* = 0.2). Among the 199 females assessed only during the intervention, mean discretionary salt intake (8.3 ± 3.7 g/d) did not differ from that of females observed during both periods (*P* = 0.3).

**Conclusions:**

Providing DFS-IoFA at no cost did not affect discretionary salt intake among participants in this trial.

This trial was registered at ClinicalTrials.gov as NCT06223854.

## Introduction

Universal salt iodization is a highly successful public health strategy to reduce iodine deficiency [1]. Mandatory salt iodization was initiated in Ethiopia in 2011, and nearly all households now use iodized salt [2]. The Ethiopian government is now considering mandatory fortification of iodized salt with folic acid to reduce the high prevalence of folate insufficiency among women of reproductive age and related neural tube defects in their offspring [2,3]. However, excessive salt intake is a major risk factor for cardiovascular diseases [4,5], and there are concerns that providing free or subsidized fortified salt or promoting its nutritional benefits could inadvertently increase discretionary salt intake.

We recently completed a community-based trial to assess the impact of double-fortified salt containing iodine and folic acid on females’ folate and iodine status (the DFS-IoFA Trial) [6]. During the 6-mo trial, we distributed iodized salt with or without folic acid bi-weekly at no cost to the households of study participants. This provided an opportunity to assess whether free distribution of fortified salt affected the participants’ discretionary salt intakes. Therefore, the objective of this analysis was to evaluate the effect of providing refined fortified salt at no cost during the intervention on discretionary salt consumption.

## Methods

### Study design, research site, participant entry criteria, and intervention

This study is a secondary analysis of data from a community-based, household-randomized, 3-arm dose–response trial conducted in Ethiopia. The parent trial evaluated the effects of 2 levels of folic acid fortification of iodized salt, compared with iodized salt alone, on biomarkers of folate and iodine status and on the occurrence of any adverse effects of the DFS-IoFA salts. The study protocol and main outcome of the study have been published [6,7], as have the results of preliminary formative research conducted before the trial, in which we measured discretionary salt intakes among 100 females in the study site [8,9].

The project was completed in 2 rural and 2 semiurban communities in the Oromia region of Ethiopia [8]. The study participants were nonpregnant females 18 to 49 y of age who provided informed consent. Eligibility criteria included current use of long-acting contraceptives, intention to remain in the community for ≥6 mo, and willingness to use the study salt exclusively for meal preparation and seasoning of individual dishes. Exclusions were acute or chronic illnesses that might affect dietary intake or folate status, use of medications that interfere with folate metabolism, and the presence of hypertension or medically prescribed salt restriction. For the current analyses, we included all females who participated in ≥1 observed weighed food record (WFR) during the trial.

During the intervention period, we provided two 500 g canisters of randomly assigned study salt to 360 participants’ households every 2 wk over a 26-wk period. The salts contained 99, 36, or 0 μg folic acid per gram salt to provide ∼600, ∼200, or 0 μg folic acid/d, respectively. All salts contained 32 ppm iodine as potassium iodate. We counseled the participants to use only the salt provided by the study for all food-related applications during the trial.

### Dietary assessments

Among the 360 participants randomly divided and allocated to 3 groups in the parent trial, as shown in the CONSORT diagram [7], 342 completed WFR during the intervention period and were included in this secondary analysis. Eighteen participants were unavailable for the WFRs because of scheduling conflicts or early withdrawal from the trial. Specially trained data collectors (“field dietitians”) conducted full-day WFRs throughout the ∼6-mo intervention period. WFRs were scheduled using a convenience-based approach, ensuring that each participant completed ≥1 WFR day during the intervention period.

Of the 342 participants, a convenience sample of 120 participants completed a second WFR ∼2 wk after the first one to estimate within-individual (day-to-day) variability in intake. The repeat WFRs were conducted among participants from all 3 intervention arms.

A total of 143 participants in the WFRs during the intervention also completed full-day WFRs before the intervention, during which they used self-procured salt, thus allowing for paired comparisons of their intakes before and during the intervention. These participants were selected for the preintervention dietary assessment before randomization to the study arm. Among the 342 participants assessed during the intervention, 199 did not participate in the WFRs before the intervention.

The field dietitians conducted the full-day WFR as previously described [6]. Briefly, they observed food preparation and consumption in the participants’ homes; and they weighed all ingredients to 0.1 g precision, including salt and salt containing, home-prepared spice mixtures added during cooking, as well as any salt added at the time of consumption. The dietitians recorded the total weight of each preparation before and after cooking, the portions served, and any leftovers. Consumed amounts were expressed as a percentage of the total postcooking weight of each preparation to calculate the amounts of each item consumed. Discrete food items, such as fruit or biscuits, were weighed separately. The next morning, dietitians recorded any nighttime intake using local utensils and a dietary scale; nighttime consumption occurred on only 1.5% of observation days.

### Study variables

The primary outcome for the current analyses is discretionary salt intake, defined as the amount of salt added during cooking or at mealtime (excluding intrinsic food sodium and salt in processed foods) and consumed by the participant, based on the observed WFRs. The definitions of outcome variables and potential covariates are provided in the statistical analysis plan for the intervention trial (https://osf.io/9pc2b) and in the study protocol [6].

### Sample size calculations

The parent trial’s sample size was based on expected changes in red blood cell folate after folic acid fortification of iodized salt [6,7]. For each of the current analyses, we calculated detectable differences in discretionary salt intakes before and during the intervention and for subsets who participated in different phases based on the available sample sizes, observed SDs, and chosen significance level (Table 1). For example, the comparison of observed discretionary salt intakes before and during the intervention among the 143 participants studied during both periods could detect intake differences ≥1.3 g/d, and the comparison of estimated usual intakes during the intervention for these 143 participants compared with the 199 participants studied only during the intervention could detect intake differences ≥0.6 g/d, considering a probability of type 1 error of 0.05 and type 2 error of 0.20 for all comparisons.

**TABLE 1:** Statistical power calculations (minimum detectable differences) for comparison of total discretionary salt intake by study period and participant subsets

Study objective	Available sample sizes for comparison		SD	Minimum detectable difference in salt intake		Statistical test
	N1	N2		Effect size (SD)	g/d	
Comparison of observed mean total discretionary salt intake preintervention vs. during intervention (same individuals)	143	143	5.3	0.24	1.3	Paired *t*-test
Comparison of mean usual total discretionary salt intake preintervention vs. during intervention (all participants)	143	342	1.8	0.28	0.5	Independent *t*-test
Comparison of mean usual total discretionary salt intake between participants who completed both preintervention and during-intervention WFRs and those who completed only during-intervention WFRs	143	199	1.8	0.31	0.6	Independent *t*-test

All detectable differences calculated for type 1 error probability = 0.05, type 2 error probability = 0.20.

Abbreviation: WFR, weighed food record.

### Data processing and statistical analysis

We conducted 4 analyses. First, we compared mean observed discretionary salt intake before and during the intervention (1-d WFR) among the 143 participants with data from both periods using paired *t*-tests. Second, we assessed mean usual preintervention salt intakes for these 143 participants compared with those of all 342 females who completed WFRs during the intervention. Third, we compared the mean usual salt intakes during the intervention for participants who did or did not complete WFRs before the intervention. Finally, we compared the mean usual salt intakes during the intervention by trial arm. Independent *t*-tests were used to compare intake across independent participant groups, and 1-way analysis of variance (ANOVA) was used to compare total preintervention salt consumption by self-selected salt type (coarse salt compared with refined salt) and across intervention arms during the study.

The assessments of usual discretionary salt intake were completed using the National Cancer Institute (NCI) method, which adjusts for intraindividual, day-to-day intake variability, implemented through SIMPLE macros in SAS [10]. Results are reported as mean usual intakes and SDs, adjusted for age, illness status, feast and fasting days, and weekends.

For preintervention and during the intervention periods, group-specific 95% confidence intervals for salt intake were derived from 200 bootstrap replicates using SEs from the NCI method. As WFRs before the intervention were completed only once for each participant, the within-to-between person variance ratio could not be estimated separately for the preintervention studies. Therefore, we applied the within-to-between person variance ratio estimated from the WFRs conducted during the intervention to adjust the preintervention distribution of usual salt intake. Comparisons of usual salt intakes across participant groups were conducted using bootstrap SEs and assessed using unequal variance *t*-tests. All statistical tests were 2-sided, and *P* values < 0.05 were considered statistically significant. Data were analyzed using SAS version 9.4 (SAS Institute Inc.), STATA version 17 (StataCorp LLC), and R version 4.4.0 (R Foundation for Statistical Computing).

### Ethical considerations

The Institutional Review Board (IRB) of the Ethiopian Public Health Institute approved the study protocol (#494-023). The University of California, Davis IRB exempted the study from review because University of California Davis only received deidentified data for analysis. The parent study was registered on ClinicalTrials.gov (NCT1834465-1).

## Results

Salt intake data were available for a total of 342 females who participated in the intervention trial and ≥1 WFR. The overall mean age of the participants was 30.4 ± 6.8 y (Table 2). Approximately one-third of the females had no formal education, most did not work outside the home, and nearly half reported some degree of food insecurity. Before the intervention, among the 143 participants who completed WFRs, 66.3% consumed coarse salt exclusively, 13.4% consumed refined salt exclusively, and 20.3% consumed a combination of both.

**TABLE 2:** Characteristics of study participants (*N* = 342)

Variable	Mean ± SD or *N* (%)
Age (y)	30.4 ± 6.8
Education	
No formal education	116 (33.9)
Any primary	125 (36.6)
Primary completed	60 (17.5)
Secondary completed and higher	41 (12.0)
Religion	
Orthodox Christian	290 (84.8)
Protestant	40 (11.7)
Muslim	9 (2.6)
Traditional	3 (0.9)
Occupation	
Not working outside the home	285 (83.3)
Trader	33 (9.7)
Farmer	10 (2.9)
Professional	10 (2.9)
Other^1^	4 (1.2)
Marital status	
Married	311 (90.9)
Divorced/separated	22 (6.4)
Single	5 (1.5)
Widowed	4 (1.2)
Household size	4.2 ± 1.5
Food insecurity	
None	187 (54.7)
Mild	46 (13.4)
Moderate	67 (19.6)
Severe	42 (12.3)
Systolic blood pressure (mm Hg)	109 ± 10
Diastolic blood pressure (mm Hg)	77 ±7
Weight (kg)	53.9 ± 9.8
Height (cm)	158.8 ± 5.4
BMI (kg/m^2^)	21.4 ± 3.5
BMI category	
Normal	205 (59.9)
Underweight	80 (23.4)
Overweight/obese	57 (16.7)

1 The category “other” refers to artisans and daily laborers.

The total observed discretionary salt intake before the intervention was 8.7 ± 4.3 g/d, of which 6.8 ± 5.1 g/d was consumed as coarse salt (Table 3). Total discretionary salt intakes did not differ by salt type before the intervention (ANOVA, *P* = 0.2). Notably, 97.8% of the participants consumed more than the WHO-recommended maximum intake amount of 5 g/d before the intervention.

**TABLE 3:** Observed discretionary salt intakes (g/d) among a subset of females who participated in weighed food records (WFRs) during both the preintervention and intervention periods or just during the intervention period, by study period and participant subsets

Salt type	Study period and participant subsets		
	Preintervention; participants in WFRs during both periods (*n* = 143)	During intervention, initial WFR; participants in WFRs during both periods (*n* =143)	During intervention, initial WFR; participants in WFRs just during intervention (*n* = 199)
Total salt (g/d)	8.7 ± 4.3	8.8 ± 4.0	8.3 ± 3.7
Self-procured coarse salt (g/d)	6.8 ± 5.1	0.4 ± 1.1	0.2 ± 0.6
Self-procured refined salt (g/d)	1.8 ± 5.1	0.3 ± 1.5	0.3 ± 1.5
Study salt (g/d)	0	8.1 ± 4.1	7.7 ± 3.8

Results are presented as mean ± SD.

There were no statistically significant differences in total discretionary salt intakes by study period or participant subsets; *P* values range from 0.19 to 0.83.

The 342 participants who completed WFRs during the intervention consumed 8.4 ± 3.8 g/d during the observation days, of which 7.8 ± 3.8 g/d (92%) was the study salt (Table 3). Overall, 99.2% of participants had a salt intake exceeding the WHO-recommended maximum intake amount of 5 g/d during the intervention. Among the 143 participants who completed preintervention WFRs, their observed total discretionary salt intake during the intervention was 8.8 ± 4.0 g/d, which did not differ significantly from their preintervention intake (*P* = 0.8). Among participants who did not complete the preintervention assessments (*N* = 199), the mean observed discretionary salt intake during the intervention was 8.3 ± 3.7 g/d, which did not differ significantly from that of participants who were assessed during both periods (*P* = 0.6).

**FIGURE 1:**
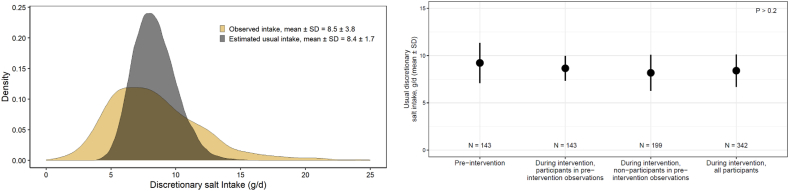
Distribution of observed and estimated usual discretionary salt intakes during the intervention period for all participants (A), and mean estimated usual discretionary salt intakes by study period and participant subsets (B). (A) Distribution of observed discretionary salt intakes and estimated usual intakes during the intervention among all participants (*N* = 342), with usual intakes derived using the NCI method. Observed intake values are shown to illustrate the distribution of measured daily intakes, whereas the estimated usual intake distribution adjusts for within person variability. (B) Mean estimated usual discretionary salt intake by study period and participant subsets, including 143 participants with both preintervention and during-intervention observations, and 199 with during-intervention observations only. Estimates are based on the NCI method for usual intake, with group comparisons conducted using *t*-tests. Abbreviations: NCI, National Cancer Institute.

During the intervention, the mean usual discretionary salt intake for all 342 participants was 8.4 ± 1.7 g/d (Figure 1). The percentage of total variance because of within-individual (day-to-day) variability during the intervention was 79.4%. This percent was applied to the before-intervention intake data to estimate the participants’ mean usual intake before the intervention, which was 9.2 ± 2.1 g/d among the 143 females studied before the intervention (Figure 1). Their mean usual preintervention discretionary salt intakes did not differ significantly from the mean usual intakes of all participants during the trial (*P* = 0.2, Figure 1), and their mean usual intakes during the trial did not differ from the mean usual intakes of females who did not participate in the preintervention studies (*P* = 0.3). As reported in the report of the parent study, salt intakes during the intervention did not differ by study arm [7].

Additional analyses showed that discretionary salt intake from before to during the intervention did not differ by food insecurity status, indicating that the intervention’s effect on salt intake was consistent across these subgroups (*P* = 0.4). Changes in discretionary salt intake from before to during the intervention did not differ by the self-selected type of salt used before the intervention (*P* = 0.6).

WFRs were distributed across the intervention period (mean study day ± SD: 86.2 ± 32; range: 18–151). The mean study day did not differ between participants with WFRs collected both before and during the intervention and those with only during the intervention WFRs (*P* = 0.7), nor across study arms (*P* = 0.1). Study day was significantly associated with discretionary salt use (*β* = −0.13/d, *P* < 0.001). However, the magnitude of this association was small and explained a limited proportion of variability in intake (*R*^2^ = 0.05). There was no overall difference in discretionary salt use between intervention groups after adjustment for study day (*P* = 0.4).

Exploratory analysis indicated that the proportion of total salt consumed during the intervention that was not the study salt did not differ significantly between intervention arms or for those who participated in the preintervention WFRs (Table 3).

## Discussion

This secondary analysis of data from the DFS-IoFA trial indicates that providing fortified, refined iodized salt at no cost to low-income Ethiopian females did not affect their discretionary salt consumption despite the fact that we provided the salt for free and informed the participants that it may have contained an additional nutrient. The overall mean usual discretionary salt intakes among study participants, both before and during the intervention, ranged between 8 and 9 g/d, which is consistent with previous national-level estimates of total daily salt intakes among females in Ethiopia (7.4 ± 1.6 g/d), based on random spot urine collections [11], and 9.3 ± 4.9 g/d usual discretionary salt intake based on dietary recall histories [12]. During the intervention period >95% of the salt consumed was the salt provided by the study, regardless of the type of salt previously consumed in the home. This consumption pattern during the trial reconfirms the previously reported high acceptability of DFS-IoFA [13]. Salt intakes during the trial did not differ among participants who did or did not participate in the preintervention observations. These results indicate that discretionary salt consumption remained consistent, and providing fortified salt at no cost did not increase consumption. This finding is reassuring for large-scale salt iodization programs in settings where additional fortificants and possible subsidies of fortified salt are being considered.

In this study, we observed a small but statistically significant decline in discretionary salt use over the intervention period. This may reflect higher intake in the early phase as households adapted to the study salt and initial preparation of salt- and spice-containing mixtures, followed by more stabilized cooking practices over time. However, the association was weak and explained limited variability in intake, suggesting minimal influence of timing on overall estimates. Importantly, adjustment for study day did not materially change comparisons between intervention groups, supporting the robustness of the primary findings.

Strengths of this study are the use of directly observed WFRs to measure discretionary salt intakes and repeated measurements among some of the participants, which enhances the accuracy of discretionary salt intake estimates. In addition, the application of the NCI method to estimate usual salt intakes improves precision by accounting for both within- and between-person variability. One possible limitation is the fact that the study was conducted in a single region of Ethiopia, so the external validity of these findings remains uncertain.

In conclusion, discretionary salt intake did not differ significantly across preintervention and during-intervention time points, between participant groups, or by study arm, indicating that the provision of fortified, refined iodized salt at no cost was not associated with an increase in discretionary salt intake among females in rural Ethiopia. Discretionary salt intake remained well above WHO-recommended limits, with nearly all of the participants exceeding intakes of 5 g/d. This finding highlights the need for complementary salt reduction strategies alongside salt fortification programs in Ethiopia and other similar settings.

## Author contributions

The authors’ responsibilities were as follows – DN, MF, IA: conducted the research and supervised project implementation; DN: analyzed the data and co-wrote the manuscript; CA: performed the statistical analysis; MW, HM: supervised project implementation; CMM, MT, KHB: designed the research and supervised project implementation; HM: provided essential materials; KHB: co-wrote the manuscript; and all authors: read and approved the final manuscript.

## Data availability

The protocol for the parent study and the statistical analysis plan for the current study (https://osf.io/9pc2b) are publicly available. Final databases with variable names and definitions and deidentified individual participant information have been archived at the Ethiopian National Data Management Center at Ethiopian Public Health Institute (https://ndmc.ephi.gov.et/) and will be made available to scientists from other institutions after publication. The metadata are publicly available at https://doi.org/10.7910/DVN/JZCF2N. Individuals who request access to the data set will be required to describe in writing the objectives of their analyses, the variables requested, and any plans for collaboration with the core research team.

## Funding

This study was supported by Nutrition International (Project No. 2284), with financial support provided by the Gates Foundation (INV-037989).

## Declaration of generative AI and AI-assisted technologies in the writing process

The authors declare that no generative AI or AI-assisted technologies were used in the writing of this manuscript.

## Conflict of interest

The authors report no conflicts of interest. KHB is a part-time consultant to the Gates Foundation but had no decision to fund this work. HM is employees of Nutrition International. He didn't recieve monetary and non-monetary compensation from the Gates foundation.
